# The Enhanced Immune Protection in Chinese Mitten Crab *Eriocheir sinensis* Against the Second Exposure to Bacteria *Aeromonas hydrophila*

**DOI:** 10.3389/fimmu.2019.02041

**Published:** 2019-08-28

**Authors:** Jingjing Wang, Bin Yang, Weilin Wang, Xiaorui Song, Qiufen Jiang, Limei Qiu, Lingling Wang, Linsheng Song

**Affiliations:** ^1^Marine Science and Engineering College, Qingdao Agricultural University, Qingdao, China; ^2^Key Laboratory of Experimental Marine Biology, Institute of Oceanology, Chinese Academy of Sciences, Qingdao, China; ^3^Laboratory for Marine Fisheries Science and Food Production Processes, Qingdao National Laboratory for Marine Science and Technology, Qingdao, China; ^4^Liaoning Key Laboratory of Marine Animal Immunology, Dalian Ocean University, Dalian, China; ^5^Dalian Key Laboratory of Aquatic Animal Disease Prevention and Control, Dalian Ocean University, Dalian, China; ^6^Liaoning Key Laboratory of Marine Animal Immunology and Disease Control, Dalian Ocean University, Dalian, China

**Keywords:** immune priming, *Eriocheir sinensis*, *Aeromonas hydrophila*, phagocytosis, immune protection

## Abstract

Accumulating evidences suggest that the enhanced immune responses and increased protection against bacteria-induced mortality can be initiated after the primary exposure to various microbial communities and their components in various organisms including commercially valuable crustaceans. In the present study, the survival rate and immune responses of Chinese mitten crab *Eriocheir sinensis* were determined after an immune priming (IP) with formalin-killed *Aeromonas hydrophila* and an immune challenge (ICH) with the same but live pathogen (Ah group). A group in which the animals received a salt injection prior to challenge was maintained as control (Ns group). In the present study, it was shown that an IP with killed *A. hydrophila* can significantly protect the crabs against the ICH with a lethal dose of the live pathogen. The increased survival was associated with elevated rate and duration of phagocytosis. The antibacterial activity of the serum was significantly increased in Ah group compared to that in Ns group. Significant changes of phenoloxidase (PO) activities were also found between Ah and Ns group but not in Ah group between IP and ICH. No significant changes of lysozyme were found in Ah and NS group during the whole experiment except 3 h after IP. In addition, the levels of transcripts and protein of Dscam were increased in hemocytes of the crabs from Ah group. All the results suggested that a primary immune priming with a particular killed pathogen could induce an enhanced immunity in crabs when they were encountered secondly with the same live pathogen. The evidences of elevated immune protections in crabs would contribute to better understand the mechanism of immune priming in invertebrates.

## Introduction

An effective immune system is vital for a host to minimize the fitness costs in response to viral, bacterial, and parasite infections. The invertebrate immune system comprises humoral and cell-mediated immunity components (1). The main cellular immune reactions include phagocytosis, nodule formation (nodulation) and encapsulation, which are thought to be the key players in protecting hosts from invading pathogens (2). Among the cellular immune responses, phagocytosis is considered as an evolutionarily conserved and complex process. The immune cells involved in phagocytosis function through engulfing and destructing pathogens. The humoral immunity consists of lysozyme, phenoloxidase, a variety of antimicrobial peptides (AMPs) and many others (3). AMPs are mainly cationic peptides with sequence diversity which endues them with a broad range of activities against microorganisms (4). Prophenoloxidase (proPO) activating system includes cascade of enzymes and other proteins located in the granular and semigranular haemocytes exerting effects on phagocytosis, cell to cell adhesion, and the formation of melanin deposits (5). It was considered as an ancestral form of a vertebrate defense pathway, possibly, an invertebrate “equivalent” or forerunner of the alternate pathway of vertebrate complement due to observations of the biochemical character and functional properties of the proPO system (6). Lysozyme widely exists in plants, invertebrates and higher animals, and is a broad-spectrum antimicrobial effector molecule. In addition, lysozyme can also induce and regulate the synthesis and secretion of the other immune factors, and cooperate with other immune factors for immune defense.

Although the invertebrate immune system has been considered to be non-specific, accumulating evidences suggest that its response against repeated pathogen infection is of both memory and specificity (7–9), but the priming effect is not universal across all bacterial strains (10). The improved survival and immune responses after secondary exposure to a previously encountered pathogen in invertebrates is now called “immune priming” (11) or “immunological memory” (12), which is defined as “the ability to store and recall information on previously encountered microbial communities or their components” (13). Some evidences have demonstrated that the cellular immune reactions like phagocytosis and some humoral factors such as antimicrobial peptide (AMP) play important roles in the immune priming in invertebrates (14–16). The specificity of invertebrate immunity is suggested to be determined by synergistic interactions among these immune components (17).

As invertebrates, arthropods are considered to have some forms of immune memory (8), for which various immunological molecules and reactions have been reported to be involved. These processes include the Down syndrome cellular adhesion molecule (Dscam) (18, 19), phagocytosis (14), antimicrobial peptides (AMPs) secretion (16, 17), hemocyte proliferation (20), transcriptional response (21), and melanisation based on proPO (22). Especially, Dscam is the most important candidate gene contributing to specific immune priming in insects and crustaceans (18, 19). It has been reported to be involved in general and specific innate immune responses in many arthropod species (23). Dscam can significantly enhance elimination of bacteria via phagocytosis in crabs. Pathogen elimination by Dscam is achieved through bacteria-specific binding and specific interactions with membrane-bound Dscam as a phagocytic receptor (24). In our previous report, the Dscam in crab (*Eriocheir sinensis*) was predicated to produce at least 53,196 alternative combinations, which was possibly enabling the animals to mount specific innate immune responses (25).

Chinese mitten crab *E. sinensis* (Arthropoda, Crustacea, Varunidae) owns critically evolutional significance and economic importance in aquaculture. The frequent outbreaks of diseases have caused drastic mortality and catastrophic economic losses in the species. The knowledge about the persistence of immune priming in *E. sinensis* is constructive to the disease management strategies in aquaculture. A gram-negative bacteria *Aeromonas hydrophila* is widely distributed in various water bodies in nature and is pathogenic for both human and animals. It is considered as not only an emerging pathogen responsible for gastro-enteritis and skin infections in human, but also a well-established pathogen involved in a range of diseases, including motile aeromonad septicemia and red sore disease in fish (26) and ascites disease in crabs. In the present study, formalin-killed *A. hydrophila* was used to prime the immune system of the crabs against a challenge with the same but live pathogen (Ah). The crabs that received a salt injection prior to challenge were maintained as control (Ns). The main objectives were (1) to investigate the survival rate of crabs after challenge with a lethal dose of live *A. hydrophila* when crabs were pre-treated with formalin-killed *A. hydrophila* or not, (2) to analyze the cellular immune reactions of phagocytic activity and the changes of total hemocyte count, and the humoral immune reactions (antibacterial activity, PO, and lysozyme activities) in the hemolymph supernatant of the crabs from Ah and Ns groups, and (3) to detect the mRNA expression alternations of immune-related genes (ALF, crustin, proPO, and Dscam), and protein levels of Dscam in Ah and Ns groups, hopefully providing more information to understand the phenomenon and mechanisms of immune priming in crustacean.

## Materials and Methods

### Animals and Bacteria

Adult Chinese mitten crabs (50 g) were collected from a local farm in Qingdao, Shandong province, China, and maintained in fresh water for 1 week before processing. During all the experiment, the crabs were fed with commercial feed (Haida Co., Ltd. in Jiangsu Province, China) once a day after every water exchange. All experiments involving animals reported in this study were approved by the Ethics Committee of the Institute of Oceanology, Chinese Academy of Sciences.

Gram-negative bacteria *A. hydrophila* kindly provided by Dr. Li Sun were used in this experiment. *A. hydrophila* were incubated in LB medium at 28°C to logarithmic growth phases. The pathogenicities (LD_50_ at 24 h) of live *A. hydrophila* were determined by stimulating the crabs with serial dilutions of bacteria. The bacteria dose for both injections was OD_600_ = 0.2 (~6 × 10^7^/mL). *A. hydrophila* used for the immune priming (IP) were inactivated with formalin at a final concentration of 0.5% for 12 h. The bacterial cells were harvested by centrifugation at 8,000 rpm, 4°C for 5 min, washed three times with sterilized 0.85% normal saline, and re-suspended in the same saline at OD_600_ = 0.2 for the following experiments. Live *A. hydrophila* used for immune challenge (ICH) was also re-suspended in the same saline at OD_600_ = 0.2.

### Experimental Design

#### Group of Treatments

**Figure 1:**
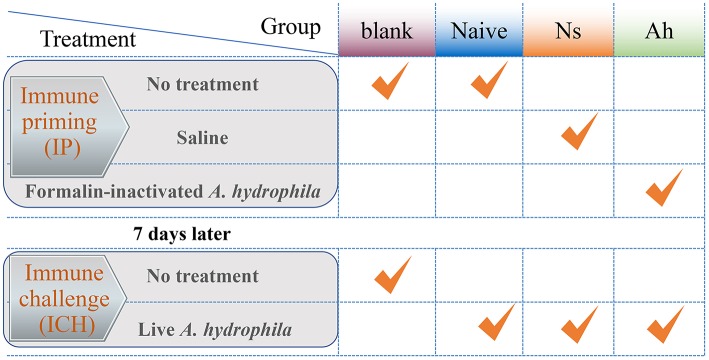
The experimental design of Chinese mitten crab *E. sinensis* receiving immune priming (IP) and lethal challenge (ICH). For survival rate in blank, Naïve, Ns and Ah group: *N* = 30; For hemolymph sampling in Ns and Ah group: *T* = 15, *N* = 6.

The experimental design was shown in Figure 1. All the experiments including survival test were repeated for three times. The experiment was divided into two stages: the first injection for immune priming (IP) and the second injection for immune challenge (ICH) after 7 days post IP. The experimental groups were designed as blank (without any treatment of IP and ICH), naïve (only an ICH with live *A. hydrophila*), Ns (IP with normal saline and ICH with live *A. hydrophila*), and Ah (IP with formalin-inactivated *A. hydrophila* and ICH with live *A. hydrophila*).

#### Immune Priming (IP)

For the IP, a total of 180 crabs received an injection of 100 μL of normal saline were employed as Ns group, and other 180 crabs received an injection of the same volume of formalin-inactivated *A. hydrophila* suspension with the effective CFU per gram crab of 1.2×10^5^ were employed as Ah group. Thirty crabs were maintained in the fresh water without any treatment and were referred to as blank. At 3, 6, 12, 24, 48, 72, and 168 h after the IP, 42 individuals of crabs from Ns and Ah groups were sampled for hemolymph collection (*N* = 6).

#### Immune Challenge (ICH)

At 168 h after the IP, all the remaining crabs from Ns group and Ah group received an injection of 100 μL of live *A. hydrophila* suspension (1.2 × 10^5^ CFU per gram crab). Forty-two crabs were sampled at 3, 6, 12, 24, 48, 72, and 168 h post-challenge for hemolymph collection (*N* = 6). Meanwhile, the crabs for the survival rate were maintained together in different groups. Each replicate was employed with 30 individuals for each treatment from blank, Naïve, Ns, and Ah group.

#### Sampling

The sampling and following analysis were performed for Ah and Ns groups. Hemolymph was collected from the cheliped using a syringe with an equal volume of anticoagulant (27 mmol L^−1^ sodium citrate, 336 mmol L^−1^ NaCl, 115 mmol L^−1^ glucose, 9 mmol L^−1^ EDTA, pH 7.0) for the phagocytosis assay and mRNA expression analysis. The supernatants of hemolymph without anticoagulant were collected by centrifugation and kept at 4°C for 3 h to measure antibacterial activity, PO and lysozyme activities from 540 samples (15 time-points ^*^6 individuals ^*^2 groups ^*^ 3 repeats).

### Total Hemocyte Counts (THC)

The hemolymph sample (about 300 μL) was fixed by adding 100 μL absolute formaldehyde, and 10 μL of the mixture was placed in a hemocytometer to measure the THC using a microscope (Olympus BX51, Tokyo, Japan).

### Phagocytosis Assay

*A. hydrophila* was incubated in LB medium at 28°C to the logarithmic growth phases and then was inactivated with formalin. After centrifugation, the bacteria cells were mixed with 0.1 mg mL^−1^ fluorescein isothiocyanate (FITC) dissolved in NaHCO_3_ (0.1 mol L^−1^, pH 9.0), and incubated in the dark on a rotator at 37°C for 3 h. The bacteria were washed three times with PBS buffer and re-suspended in 20 mL PBS at a final concentration of 10^8^ CFU/mL. The suspension of labeled bacteria was divided into 1 mL aliquots and stored at −20°C for later use.

Hemolymph samples with anticoagulant were immediately centrifuged at 800 × g at 4°C for 10 min after which the hemocytes were harvested. The hemocytes were washed and re-suspended in sterile saline at a final concentration of 10^5^ cells/mL. The cell suspension was mixed with 1 mL of FITC-labeled bacteria solution at a ratio of cell/bacteria = 1:100, and incubated in the dark at room temperature for 1 h. Then 200 μL of the cell suspension was dispensed onto the slides to allow the hemocytes to adhere at room temperature for 1 h. After being washed with saline for three times, trypan blue was added onto the slides and incubated for 15 min to quench the un-phagocytosised fluorescent bacteria. Trypan blue was washed away with saline until there was almost no blue staining anymore on the slides. After being rinsed in saline, the slides were mounted with 50% glycerin and coverslip, and stored at 4°C in the dark until examination using an Olympus BX51 fluorescence microscope. The percentage of phagocytosed cells (PR) and phagocytic index (PI) was calculated according to the formula expressed as following: PR = (phagocytic hemocytes)/(total hemocytes) × 100%; PI = average number of bacteria in phagocytic hemocyte.

### Antibacterial Assay

#### Hemolymph and Bacteria Preparation

The hemolymph without anticoagulant was centrifuged at 5000 × g at 4°C for 10 min to collect supernatant. After cultured in LB medium overnight, *A. hydrophila* was washed twice with sterile PBS, and re-suspended in the sterile PBS solution at a final concentration of 10^4^ bacteria/mL.

#### Incubation Experiment

For the antibacterial assay, 50 μL of bacteria resuspension and 50 μL supernatant, or equal volume of PBS, were incubated at room temperature with shaking for 30 min. Subsequently, 20 μL of this suspension was pipetted into a sterile 96 well plate with 200 μL of LB. The plate was incubated for 16 h at 28°C on a plate reader, and the absorbance at 600 nm was measured every half an hour. Bacteria incubated with sterile PBS solution were used as control.

#### Values of ΔΔOD

The maximum OD_600_ was recorded at the time point when the growth curve of bacteria-only control reached the plateau. The T_50_ was the half time (h) taken by the bacteria-only control to reach maximum OD_600_. The preliminary experiments revealed that the T_50_ value was 5 h for *A. hydrophila* control. The OD_600_ values at T_50_ were determined in all the test groups. To eliminate background values, the OD_600_ values at T = 0 h were subtracted for each sample as follows: ΔOD = OD_600_ (5 h) – OD_600_ (0 h). The differences of ΔOD values between control and tested samples were calculated as ΔΔOD = ΔOD_Control_ – ΔOD_Test_, and the values of ΔΔOD were employed to indicate the antibacterial activities.

### Activity Measurements of PO and Lysozyme

The previous research showed that the PO activity in serum was higher than that in HLS or plasma (data not shown) and the enzyme activity in the serum was not influenced by freezing in −80°C (27, 28). In the present study, PO activity in the supernatant, incubated at −80°C, was measured spectrophotometrically by recording the formation of dopachrome from L-DOPA (Sigma Chemical Company, MO, USA) after the supernatant was melted at 4°C. Briefly, aliquots (100 μL) of the supernatant were mixed with 100 μL L-DOPA (3 mg mL^−1^) and the OD_490_ was measured every 2 min at 490 nm for 30 min. PO activity was recorded as the maximum absorbance change over any 1 min interval (ΔOD_490_ nm/min) during the assay. A unit (U) of PO activity was defined as the amount of sample causing increase in absorbance of 0.001 per minute.

The activity of lysozyme and concentration of proteins were measured using corresponding detection kits (Jiancheng, Nanjing, China) according to the manufacturer's guidelines. The lysozyme activities were expressed as units per milligram of protein (U/mg). The total protein of each sample was determined using the Bradford method (29).

### Quantitative Real-Time PCR and Statistical Analysis

Hemolymph samples were immediately centrifuged at 800 × g at 4°C for 10 min to harvest the hemocytes, and they were stored at −80°C after adding 1 mL TRizol reagent (Invitrogen) for subsequent RNA extraction. The total RNA extraction, cDNA synthesis, primer designing for target sequences and SYBR Green fluorescent PCR amplification were all carried out according to Minimum Information for Publication of Quantitative Real-Time PCR Experiments from Sigma-Aldrich Co. The genes of Anti-lipopolysaccharride factor (ALF), crustin, proPO, and Dscam (GenBank accession no: DQ793214, GQ200832, EF493829, and JX501778) from *E. sinensis* were selected as target immune-related genes and all the primers used were listed in Table 1. The primers of Dscam used in present study were designed within Ig 4 domain, which was a constant exon. The expressions of these immune-related genes were normalized to the expression of β-actin gene for each sample and the comparative Ct method (2^−ΔΔ*Ct*^ method) (30) was used to qualify their expression levels.

**Table 1:** The primers used in the present study.

**Primer**	**Sequence (5′-3′)**	**Remark**
Cr-RTF	TGCTGCGAAGATGAACGGGAAAT	Crustin real-time PCR
Cr-RTR	CTAGTAGGAGGACACACAGGGCG	Crustin real-time PCR
Pr-RTF	CCATCCCTTCCTGCTTACCA	proPO real-time PCR
Pr-RTR	CTCCATCACAAACCCTAACGACTT	proPO real-time PCR
Al-RTF	GACGCAGGAGGATGCTAAC	ALF real-time PCR
Al-RTR	TGATGGCAGATGAAGGACAC	ALF real-time PCR
Ds-RTF	GTGGAACCCAAAGTACAGACCG	Dscam real-time PCR
Ds-RTR	AGAGTTGATGCGAAGAACAGCC	Dscam real-time PCR
Actin-F	GCATCCACGAGACCACTTACA	β-actin Real-time PCR
Actin-R	CTCCTGCTTGCTGATCCACATC	β-actin Real-time PCR

### Preparation of Dscam Antibody and Western Blotting Analysis

The polyclonal antibody of Dscam used in the present study was prepared by immuning the 6 weeks old rats with recombination crab Dscam protein for four times and verified its specificity using western blotting analysis in our previous report (25). Five mixed samples were prepared for western blotting, including blank, Ns group at 12 h after the IP and ICH, Ah group at 12 h after the IP and ICH. For each sample, the equal aliquot (60 μg/20 μL) was loaded and the western blot assay was repeated three times.

Briefly, the protein samples from crab hemocytes were separated by SDS-PAGE, and electrophoretically transferred onto a 0.45 mm pore nitrocellulose membrane at 200 mA for 5 h. The membrane was blocked with PBS containing 3% BSA at 37°C for 1 h, and incubated with antibody at 37°C for 1 h. After being washed three times with PBS containing 0.05% Tween-20 (PBS-T), the membrane was incubated with goat anti-rat Ig-HRP conjugate (Southern Biotech, diluted 1:5000 in PBS) at 37°C for 1 h. After the final three times of washing with PBS-T, the membrane was stained with Western Lightning ECL Pro (PerkinElmer), and then developed with Kodak films. Rats' pre-immune serum was used as negative control. The intensity of target proteins was obtained by scanning the gray levels of the protein band using Photoshop software and then adjusted by eliminating background values.

### Statistical Analysis

The survival rate was calculated with a Kaplan-Meier estimate followed by a log-rank test in SPSS 17.0. The other data from six individuals in each group at one time point were firstly averaged and expressed as the mean of triplicate assays with the standard error (SD), which were then analyzed by using SPSS 17.0. The data between Ah and Ns groups at each time-point were separately analyzed by unpaired Student's *t*-test. When the differences of individual time-points were significant at <5% level, i.e., the values in Ah group were significantly higher or lower than those in Ns group, a further S-N-K test was used to compare the mean values between these Ah groups with significant difference. Data with any letter means significant difference between Ah and Ns groups at this time-point (*p* < 0.05). And different letter (a, b, c) means significant difference between different Ah groups (*p* < 0.05).

## Results

### Elevated Survival Rate of Crabs After an ICH With Live *A. hydrophila*

**Figure 2:**
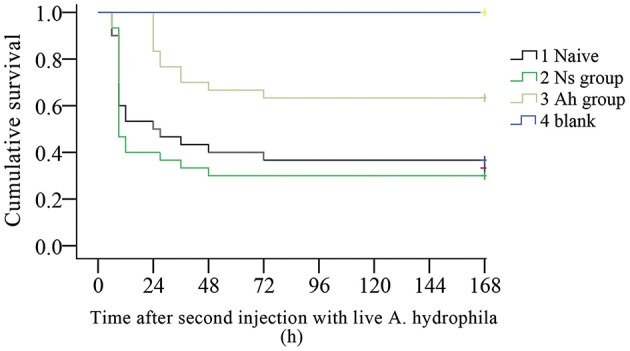
Kaplan-Meier cumulative survival following the ICH with live *Aeromonas hydrophila* after previous IP with *A. hydrophila* (Ah group) or Normal saline (Ns group) or without any previous injection (Naïve) in *E. sinensis* (*N* = 30). Blank crabs without any injections during IP and ICH were employed as control. 0–168 h means the time started after ICH. This survival experiment was repeated for three times.

The survival rates of Naïve, Ns, Ah, and blank groups (*N* = 30) were recorded by counting the dead individuals every 3 h. After the IP, no mortality was observed in four groups (data not shown). On the 7th day post IP, the animals received an ICH with a lethal dose of the live *A. hydrophila*. The mortality was observed in Ns group and Naïve group at 3 h, while late in Ah group at 24 h post ICH. The log-rank test showed a significant difference in survival rate between Ah group and three other groups after the ICH (*p* < 0.05). After the ICH, the survival rate in Ns group did not change significantly compared to that of the Naïve group (*p* > 0.05). No additional mortality was observed in Ns group after 48 h post ICH, and in Ah and Naive groups after 72 h. No crabs died in the blank group (Figure 2).

### The Changes of Total Haemocyte Count (THC) After the ICH With Live *A. hydrophila*

**Figure 3:**
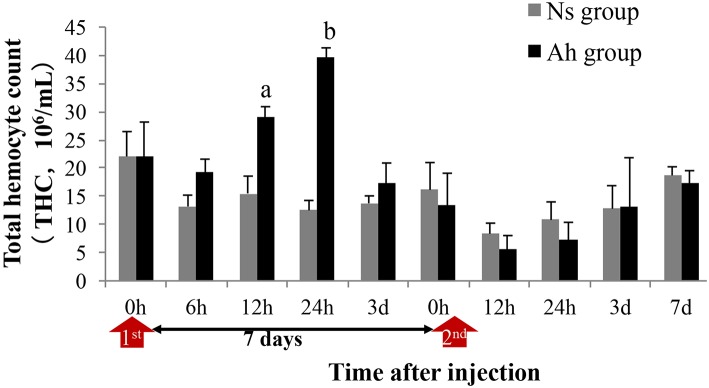
Effects of *A. hydrophila* on Total Hemocyte Counts observed under an Olympus BX51 fluorescence microscope in *E. sinensis*. Each bar represents the mean of triplicate assays with the standard error (SD) after data of six individuals in each group at one time point was firstly averaged. Red arrow indicates the 1st injection (IP) and 2nd injection (ICH). Bars with any letter mean significant difference on the THC between Ah and Ns groups at one time-point after *t*-test (*p* < 0.05). Bars with different letters mean significant difference among the Ah groups after S-N-K test (*p* < 0.05).

THC in Ah group was significantly increased (*p* < 0.05) compared to that in Ns group at 12 and 24 h after the IP (Figure 3). On the 3rd day post IP, THC levels declined and reached their initial values in Ah group. At this point, no significant difference (*p* > 0.05) was observed in the levels of THC collected from Ah and Ns groups. After the ICH, THC levels declined at 12 and 24 h in Ah group without significant differences compared to that in Ns group (*p* > 0.05; Figure 3).

### The Long Lasting Enhanced Phagocytic Activity of Hemocytes After the ICH With Live *A. hydrophila*

The phagocytic activities including the phagocytic rate (PR) and phagocytic index (PI) were determined by the observation under fluorescence microscope after an incubation of FITC-labeled *A. hydrophila* with hemocytes.

**Figure 4:**
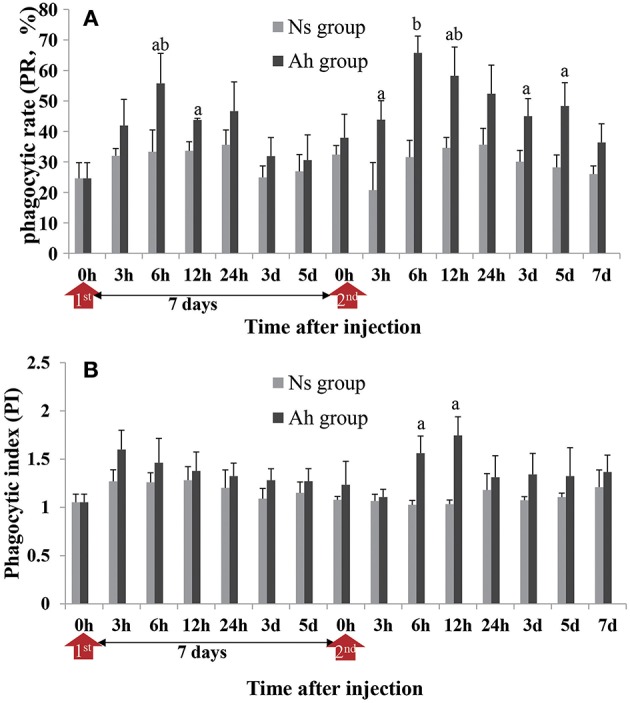
Effects of *A. hydrophila* on hemocyte phagocytic activity using the FITC labeled *A. hydrophila* observed under an Olympus BX51 fluorescence microscope. **(A)** The phagocytic rate (PR) and **(B)** phagocytic index (PI) in response to the IP with formalin-killed *A. hydrophila* and the ICH with live *A. hydrophila* in *E. sinensis*. Each bar represents the mean of triplicate assays with the standard error (SD) after data of six individuals in each group at one time point was firstly averaged. Red arrow indicates 1st injection (IP) and 2nd injection (ICH). Bars with any letter mean significant difference on the phagocytic activities between Ah and Ns groups at one time-point after *t*-test (*p* < 0.05). Bars with different letters mean significant difference among the Ah groups after S-N-K test (*p* < 0.05).

After an IP, the PR of hemocytes in Ah group peaked at 6 h (*p* < 0.05), and then significantly (*p* < 0.05) decreased, compared to Ns group, starting from 12 h post IP. After the ICH, the PR of hemocytes in Ah group was increased significantly compared to Ns group (*p* < 0.05) starting from 3 h, and maintained at the high level during 6 h to 5 days. No significant difference was observed in Ah group at 7 days after ICH compared to Ns group (*p* > 0.05; Figure 4A). The S-N-K test revealed a significant difference of PR values in Ah group between 12 h after the IP and 6 h after the ICH (*p* < 0.05; Figure 4A).

The phagocytic index (PI) was also enhanced in the Ah group after the ICH, but the alternation was less than that of PR. PI in Ah group peaked at 3 h (*p* > 0.05) after the IP, but peaked late at 12 h after the ICH (*p* < 0.05). At other time-points except 6 h after the ICH (*p* < 0.05), there was no statistical difference in phagocytic index in Ah group compared to Ns group (*p* > 0.05; Figure 4B).

### The Change of Antibacterial Activity in Hemolymph Supernatant After the ICH With Live *A. hydrophila*

**Figure 5:**
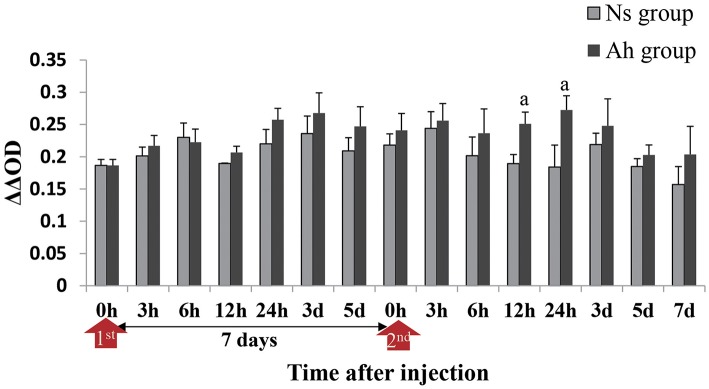
Antibacterial activities in hemolymph supernatant of crabs receiving the IP with formalin-killed *A. hydrophila* and the ICH with live *A. hydrophila* in *E. sinensis*. Bacterial growth was recorded as absorbance at 600 nm from the time (h) closest to the calculated T_50_ for the bacteria-only control after T = 0 had been subtracted. ΔΔOD = [OD _Control(T50)_ – OD _Control(0h)_] – [OD _Test(T50)_ – OD _Test(0h)_]. Each bar represents the mean of triplicate assays with the standard error (SD) after data of six individuals in each group at one time point was firstly averaged. Red arrow indicates 1st injection (IP) and 2nd injection (ICH). Bars with any letter mean significant difference on the antibacterial activities between Ah and Ns groups at one time-point after *t*-test (*p* < 0.05).

The antibacterial activity in the hemolymph supernatant from Ah group against *A. hydrophila in vitro* peaked at 3 days after the IP compared to that from Ns group (*p* > 0.05). The antibacterial activities in the hemolymph supernatant from Ah group after the ICH were significantly higher than that from Ns group (*p* < 0.05) at 24 h. There was no significant difference between Ns and Ah groups (*p* > 0.05) at other time-points (Figure 5).

### Enzyme Activities After the ICH With Live *A. hydrophila*

**Figure 6:**
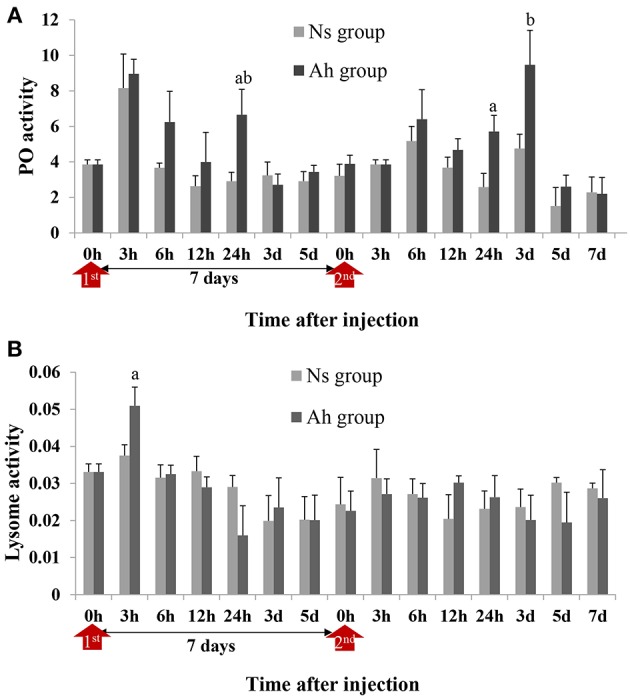
PO and lysozyme activities in hemolymph supernatant of crabs receiving the IP with formalin-killed *A. hydrophila* and the ICH with live *A. hydrophila*. **(A)** PO activity in *E. sinensis*, **(B)** Lysozyme activity in *E. sinensis*. Each bar represents the mean of triplicate assays with the standard error (SD) after data of six individuals in each group at one time point was firstly averaged. Red arrow indicates 1st injection (IP) and 2nd injection (ICH). Bars with any letter mean significant difference on PO and lysozyme activities between Ah and Ns groups at one time-point after *t*-test (*p* < 0.05). Bars with different letters mean significant difference among the Ah groups after S-N-K test (*p* < 0.05).

PO activities of crabs from Ah group were increased significantly at 24 h post IP compared to Ns group (2.28-fold, *p* < 0.05) and then decreased to the normal level after 3 days. After ICH, PO activities in Ah group were increased from 24 h (2.22-fold, *p* < 0.05) to 3 d (1.99-fold, *p* < 0.05) and then decreased further, with no significant difference (*p* > 0.05) compared to the results obtained from Ns group (Figure 6A). S-N-K test showed that there was no significant difference of PO activities in Ah group at the peak time between the IP and ICH (*p* > 0.05; Figure 6A).

Lysozyme activity of crabs from Ah group were increased significantly (*p* < 0.05) at 3 h after the IP compared to Ns group, decreased slightly after-wards, and then returned to the baseline levels after 7 days. After the ICH, lysozyme activities of crabs from Ah group did not change significantly at all time-points (*p* > 0.05) compared to Ns group (Figure 6B).

### The Alternations of mRNA Expression Levels of Immune-Related Genes

Three candidate genes involved in the process of antibacterial and PO activities, including crustin, proPO and ALF, were selected according to the previous reports (5, 31). Their mRNA expressions after the IP and the ICH with killed or live *A. hydrophila* were examined by real-time PCR to investigate the possible mechanism of the specifically enhanced humoral immune responses. The expression levels of each gene were given in fold changes compared to the expression level at 0 h (blank).

**Figure 7:**
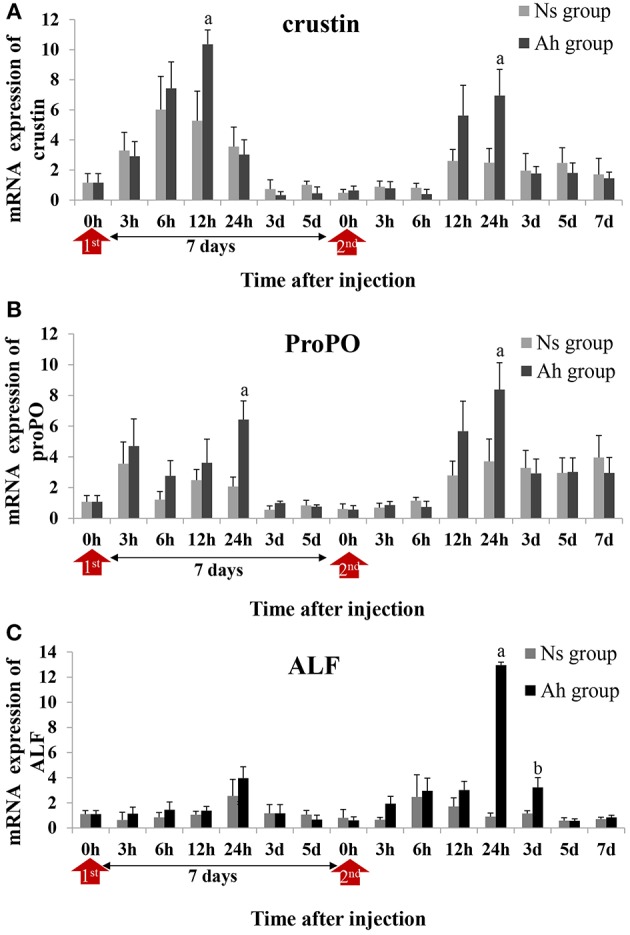
Relative expression of immune-related genes in crab hemocytes. The mRNA expressions of **(A)** Crustin, **(B)** ProPO, and **(C)** ALF were measured following the IP with formalin-killed *A. hydrophila* and the ICH with live *A. hydrophila* in *E. sinensis*. Each bar represents the mean of triplicate assays with the standard error (SD) after data of six individuals in each group at one time point was firstly averaged. Red arrow indicates 1st injection (IP) and 2nd injection (ICH). Bars with any letter mean significant difference on mRNA expression between Ah and Ns groups at one time-point after *t*-test (*p* < 0.05). Bars with different letters mean significant difference among the Ah groups after S-N-K test (*p* < 0.05).

After the IP, the mRNA expression of crustin in Ah group was significantly increased and peaked at 12 h (*p* < 0.05) compared to Ns group, which was earlier than that of proPO (*p* < 0.05) and ALF (*p* > 0.05) (peaked at 24 h) (Figures 7A–C). After the ICH, the mRNA expressions of crustin (2.8-fold, *p* < 0.05), proPO (2.3-fold, *p* < 0.05), and ALF (14.4-fold, *p* < 0.05) in Ah group were all increased and peaked at 24 h, compared to that in Ns group, and then down-regulated and returned to the original levels at 7 days (*p* > 0.05). S-N-K test showed that there was significant difference of ALF mRNA expressions in Ah group (*p* < 0.05) observed between the IP and ICH (Figures 7A–C).

### The mRNA and Protein Expression Levels of Dscam After IP and ICH With *A. hydrophila*

**Figure 8:**
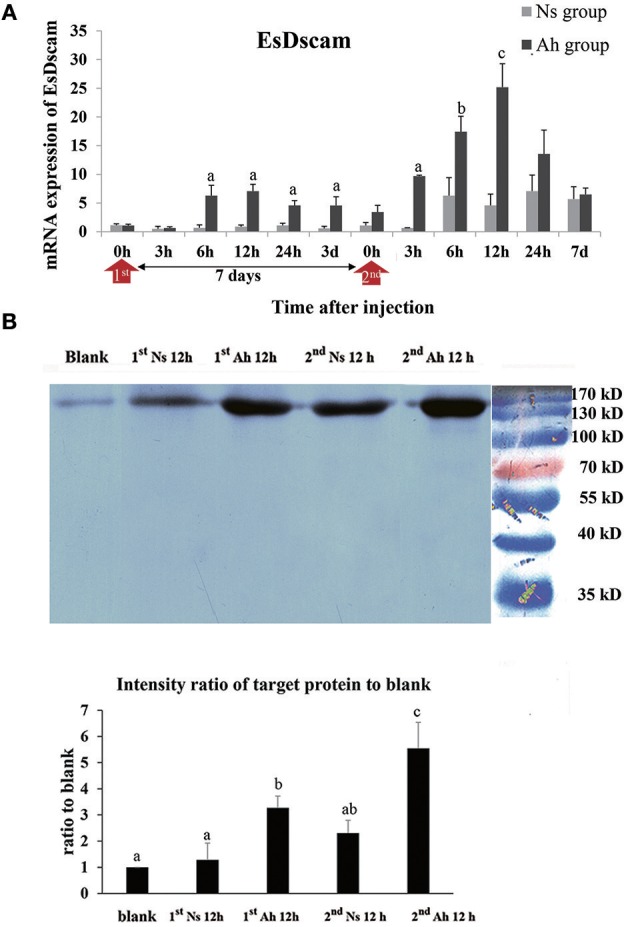
The mRNA expression and protein level of Dscam in crab hemocytes. **(A)** The mRNA expression following the IP with formalin-killed *A. hydrophila* and the ICH with live *A. hydrophila* in *E. sinensis* using real-time PCR method. Each bar represents the mean of triplicate assays with the standard error (SD) after data of six individuals in each group at one time point was firstly averaged. Red arrow indicates 1st injection (IP) and 2nd injection (ICH). Bars with any letter mean significant difference on Dscam mRNA expression between Ah and Ns groups at one time-point after *t*-test (*p* < 0.05). Bars with different letters mean significant difference among the Ah groups after S-N-K test (*p* < 0.05). **(B)** Western-blot analysis of Dscam in *E. sinensis*. Lane 1: blank group; lane 2: Ns group at 12 h after 1st injection (IP) with normal saline; lane 3: Ah group at 12 h after 1st injection with formalin-killed *A. hydrophila*; lane 4: Ns group at 12 h after 2nd injection (ICH) with live *A. hydrophila*; lane 5: Ah group at 12 h after 2nd injection with live *A. hydrophila*; lane 6: protein molecular standard. The histogram below showed the intensity ratio of target proteins to blank group after scanning the gray levels of the protein band using Photoshop software. Each bar represents the mean of triplicate assays with the standard error (SD). Bars with different letters mean significant difference among the groups after ANOVA (*p* < 0.05).

The mRNA expression of Dscam was calculated by real-time PCR. The mRNA expression level of Dscam in Ah group was up-regulated from 6 to 12 h after the IP (*p* < 0.05) and peaked at 12 h (6.3-fold, *p* < 0.05) compared to Ns group. After the ICH, the mRNA expression of Dscam in Ah group was increased at 3 h, which was 3 h earlier than that after the IP. The mRNA expression level of Dscam in Ah group after the ICH was significantly higher at 3 h (15.1-fold, *p* < 0.05), 6 h (2.8-fold, *p* < 0.05), 12 h (5.5-fold, *p* < 0.05), and 24 h (1.9-fold, *p* < 0.05) with a peak at 12 h compared to that in Ns group. There were no other notable differences in mRNA levels of Dscam between the Ns and Ah group (*p* > 0.05). The S-N-K test showed a significant difference of Dscam mRNA expression at 6, 12, 24 h after the ICH compared to those after the IP in Ah group (*p* < 0.05; Figure 8A).

The expression of Dscam protein was examined by western blotting assay. The total amount of hemocyte protein was adjusted to the same levels (60 μg/20 μL) for the western blotting analysis. A single band about 170 kDa was observed in all the detected samples, which was in accordance with Dscam. S-N-K test of the intensity ratio of target proteins to blank indicated that Dscam protein levels in Ah group at 12 h after the IP and ICH were significantly higher than those in blank and Ns group (*p* < 0.05), respectively. The intensity of Dscam in Ah group at 12 h after the ICH was even higher than that at 12 h after the IP (*p* < 0.05; Figure 8B).

## Discussion

It has been reported that the invertebrates such as cephalochordate (32), mollusca (33, 34), crustaceans (35), and insects (36) can produce a stronger immune response resulting in increased survival rate after a previous encounter with various microbial communities and their components. This phenomenon is termed as “immune priming.” The immune priming was reported to be related with several factors, including different host-parasite interactions, challenge or priming dose, and time post priming (12). In the present study, the crabs were initially immune primed (IP) with formalin-killed *A. hydrophila*, and were immune challenged (ICH) with live *A. hydrophila* with the lethal dose of LD_50_ at the 7th day post IP (Ah group). The survival rate after the ICH was recorded in order to find out the existence of immune priming in crabs. The mortality in Ah group was observed at 24 h after the ICH, which was 21 h later than that in Ns group. The log-rank test showed that the survival rate of Ah group was significantly higher than that in Ns group after the ICH. In the present study, the enhanced immune protection initiated after the primary exposure to the same strain of bacteria indicated the existence of immune priming in *E. sinensis*.

Although the phenomenon of immune priming has been observed in an increasing number of invertebrates, the detailed mechanisms at molecular and cellular level are still not well-understood. Therefore, next, we investigated the possible immunological mechanisms behind the observed phenotypes. Some evidences have demonstrated that phagocytosis plays an important role in the elevated immune protection (14, 15). For example, the phagocytosis was found to be involved in priming responses against two strains of *Bacillus thuringiensis* upon pricking infection in a crustacean species (15). In the present study, the phagocytosis against *A. hydrophila* was examined in order to explore the possible cellular mechanism of immune priming. The IP of formalin-inactivated *A. hydrophila* could induce the significant increase of PR and PI of hemocytes when they encountered the ICH of *A. hydrophila*. It was worth mentioning that the priming of *A. hydrophila* consequentially aroused persistently enhanced phagocytosis of hemocytes in crab, which could last from 3 h to 5 days after an ICH with live *A. hydrophila* accompanied by the decrease of total hemocytes number. The faster and stronger immune response of phagocytosis after the immune challenge demonstrated the potential importance of cellular response against *A. hydrophila* in this enhanced immune protection.

Besides the cellular immune reactions like phagocytosis, some humoral factors are reported to be involved in the immune responses of invertebrate. In invertebrates, the phenoloxidase (PO) system is an important constituent of the innate immune repertoire to the animals against invading pathogens. The molecules are involved in melanization, coagulation (37), phagocytosis, and promotion of bacterial agglutination and clearance (38). In addition, PO genes have been annotated in most of the arthropods, suggesting that they are probably necessary for survival. However, some controversy exists in the field, and at least in flies and mosquitoes, the successful combat of some pathogens does not seem to be directly dependent on phenoloxidase activity (5). In pancrustaceans, it has long been clear that somatically generated immune factors, such as lectins and proPO-related proteins, could not account for immune specificity or immune memory (39). In the present study, no significant difference (*p* > 0.05) was observed in PO activity of the haemolymph supernatant collected from various treatment groups (i.e., Ah, IP, and ICH). These results suggest no obvious involvement of PO activity in immune priming of crabs against *A. hydrophila*. In addition, the mRNA expression of proPO gene, which can be hydrolyzed by serine protease to produce active phenoloxidase, was also measured using real-time PCR. In accordance with the PO activity, the mRNA expression of proPO in Ah group was increased significantly at 24 h after the ICH, compared to Ns group, but no significant difference was found in Ah group compared to that after the IP. Although immune priming can result in higher expression of immune genes, which may not be translated until there is an immunological threat, based on our observations, we can conclude that PO may be an exception to this statement in mitten crabs. Similarly, the lysozyme activities of crabs from Ah group did not change significantly compared to Ns group after the ICH at all time-points. This may be because of redundancy among separate immune mechanisms, species differences or a combination thereof (5). Another humoral factor antimicrobial peptide (AMP), is reported to be involved in the antibacterial responses and immune priming of invertebrate (16). Individual immune priming in *T. molitor* is achieved through a sustained antibacterial activity, which can be active for at least 20 days after a primary immune challenge by injection of a suspension of killed Gram-positive bacteria (7, 40). In *T. molitor*, AMP exhibited enhanced antimicrobial activity at 7 day post priming (41). In the present study, the antibacterial activity was also investigated to verify its role in immune priming of crabs. When hemolymph supernatant was incubated with *A. hydrophila in vitro*, the antibacterial activity of hemolymph in Ah group was increased significantly at 24 h after ICH compared to that in Ns group. Moreover, crustin and ALF genes involved in antibacterial activity were selected to explore their mRNA expressions in Ah group after the IP and the ICH. The peak level of ALF mRNA expression in Ah group after the ICH was significantly and by 3.27-folds higher than that after the IP, while the mRNA expression of crustin was not triggered after an ICH. In a word, ALF might be involved in the *A. hydrophila*-induced immune protection in crabs, while the PO and lysozyme might not. PO activity might be negatively regulated by the enhanced antibacterial activity due to the balance of energy budget in immune priming (42, 43).

The three hallmarks of acquired immunity are immune diversity, immune specificity, and immune memory (44). The specificity of invertebrate immunity is suggested to be determined by synergistic interactions among immune components and high genetic diversity of receptors or effectors (17). Dscam has been proposed to be one of the key candidates for a somatically diversified receptor system in the crustaceans and insects (Pancrustacea), which could enable challenge-specific protection (45, 46). The immune diversity and specificity of Dscam gene through alternative RNA splicing could be an alternative way to function as vertebrate antibodies in the immune responses (45, 47). Accumulating evidence implicates that Dscam might be involved in immunity against a non-self component in long-lived crustaceans, such as crab and shrimp (24). However, the information about the evidence of Dscam being involved in the immune memory of crustaceans is still very limited (46). In our previous study, Dscam was found to produce 53,196 isoforms in *E. Sinensis* to recognize various pathogens and play an active role in immune defense (25). EsDscam isoforms were found to bind specifically with the original bacteria to facilitate efficient clearance. Furthermore, bacteria-specific binding of soluble EsDscam via the complete Ig1–Ig4 domain significantly enhanced elimination of the original bacteria via phagocytosis by hemocytes (24). In the present study, the mRNA and protein expressions of Dscam after IP and ICH with *A. hydrophila* were investigated to identify the precise role of Dscam in crab immunity. After the ICH, the mRNA expression of Dscam in Ah group was significantly increased 3 h earlier than that after the IP, and the maximum expression level was 5.5-fold higher than that in the Ns group. The Dscam protein in Ah group was found to be much more abundant at 12 h after ICH than that at 12 h after IP. It was in accordance with the opinion that Dscam exhibited a typical fast (2–6 h) immune response to pathogen-associated molecular patterns (PAMPs). However, Dscam expression (total or alternatively spliced variants) after exposure to a pathogen or parasite shows varied results across studies (45). Some studies also demonstrated that Dscam was not always induced immediately after immune stimulation. Instead, viruses and bacteria usually took more than 24 h to induce elevated Dscam levels (48, 49). Overall high expression levels are not the only indication of Dscam's role in immunity, and it now appears that the correct combination of Dscam isoforms might be more important (39). In crabs, soluble Dscam regulates hemocyte phagocytosis via bacteria-specific binding and specific interactions with membrane-bound Dscam as a phagocytic receptor. In the current study, increased levels of both EsDscam mRNA and protein, provided evidences for the possible role of this molecule in phagocytosis and bacterial clearance. But to what extent EsDscam may regulate phagocytosis remains unclear. During this phagocytosis process, many molecules including secreted molecules, putative transmembrane receptors, and some intracellular molecules have been identified to be regulators of phagocytosis (50). In the present study, PR in Ah group began to increase significantly at 6 h after the IP and 3 h after the ICH compared to Ns group, respectively. The significantly higher mRNA expression of EsDscam in Ah group appeared at the same time. It showed that expression of EsDscam mRNA didn't increase earlier than phagocytosis in the present study, which may suggest that EsDscam was not the only or predominant factor for phagocytosis despite its role in promoted phagocytosis in crabs. Collectively, Dscam was proposed as a key candidate for a somatically diversified receptor system and might contribute partly to enhanced phagocytosis and bacteria-specific binding in crustacean and pancrustacean (51).

Individual immune priming may rely on three types of responses (52). First, it may involve a sustained response, corresponding to the long-lasting upregulation of the same immune effectors after the initial immune priming, just as the elevated phagocytosis in the present study. Second, a recalled response results in a faster and stronger response after a secondary infection in a way that is reminiscent of the vertebrate acquired immune response, which was in accordance with recalled higher phagocytosis, antibacterial activity, mRNA expression of ALF and Dscam after an ICH in our present results. Third, priming may induce an immune shift, involving different immune effector systems during the primary and the secondary immune responses (42). In our study, humoral and cellular immune system was proved to respond to both immune priming and immune challenge. Many researchers are interested in and now working on the mechanisms underpinning trained immunity in invertebrate. Besides the factors studied in present research, epigenetic programming such as DNA methylation and histone modifications at specific immune-related loci is likely to be involved in the mechanisms leading to trained immunity (53). However, these mechanistic details need further verification and there is still a long way to go.

In conclusion, higher survival rate in Ah group with an IP of killed *A. hydrophila* and an ICH of live *A. hydrophila* provided the evidence of immune priming in *E. sinensis* against *A. hydrophila*. The long-lasting and higher phagocytosis, specifically enhanced antibacterial activity and highly expressed Dscam were suggested to be the possible mechanisms for these elevated immune protection.

## Data Availability

All datasets generated for this study are included in the manuscript and/or the supplementary files.

## Ethics Statement

All animal-involving experiments of this study were approved by the Ethics Committee of Institute of Oceanology, Chinese Academy of Sciences.

## Author Contributions

JW, LW, and LS conceived, designed the experiments, and wrote the manuscript. JW, BY, and WW developed the methodology and performed the experiments. JW, WW, and QJ analyzed the data. XS, QJ, and LQ contribute to the discussion. All the authors read and approved the final manuscript.

### Conflict of Interest Statement

The authors declare that the research was conducted in the absence of any commercial or financial relationships that could be construed as a potential conflict of interest.
